# Information and Communication Technology to Support Self-Management of Patients with Mild Acquired Cognitive Impairments: Systematic Review

**DOI:** 10.2196/jmir.2275

**Published:** 2012-11-19

**Authors:** Aboozar Eghdam, Jeremiah Scholl, Aniko Bartfai, Sabine Koch

**Affiliations:** ^1^Health Informatics Centre (HIC)Department of Learning, Informatics, Management and Ethics (LIME)Karolinska InstitutetStockholmSweden; ^2^Division of Rehabilitation MedicineDepartment of Clinical Sciences Danderyds HospitalKarolinska InstitutetStockholmSweden

**Keywords:** Assistive technology, Classification, Disability, Information and Communication Technology, Mild Acquired Cognitive Impairments, Self-management, Traumatic Brain Injuries

## Abstract

**Background:**

Mild acquired cognitive impairment (MACI) is a new term used to describe a subgroup of patients with mild cognitive impairment (MCI) who are expected to reach a stable cognitive level over time. This patient group is generally young and have acquired MCI from a head injury or mild stroke. Although the past decade has seen a large amount of research on how to use information and communication technology (ICT) to support self-management of patients with chronic diseases, MACI has not received much attention. Therefore, there is a lack of information about what tools have been created and evaluated that are suitable for self-management of MACI patients, and a lack of clear direction on how best to proceed with ICT tools to support self-management of MACI patients.

**Objective:**

This paper aims to provide direction for further research and development of tools that can support health care professionals in assisting MACI patients with self-management. An overview of studies reporting on the design and/or evaluation of ICT tools for assisting MACI patients in self-management is presented. We also analyze the evidence of benefit provided by these tools, and how their functionality matches MACI patients’ needs to determine areas of interest for further research and development.

**Methods:**

A review of the existing literature about available assistive ICT tools for MACI patients was conducted using 8 different medical, scientific, engineering, and physiotherapy library databases. The functionality of tools was analyzed using an analytical framework based on the International Classification of Functioning, Disability and Health (ICF) and a subset of common and important problems for patients with MACI created by MACI experts in Sweden.

**Results:**

A total of 55 search phrases applied in the 8 databases returned 5969 articles. After review, 7 articles met the inclusion criteria. Most articles reported case reports and exploratory research. Out of the 7 articles, 4 (57%) studies had less than 10 participants, 5 (71%) technologies were memory aids, and 6 studies were mobile technologies. All 7 studies fit the profile for patients with MACI as described by our analytical framework. However, several areas in the framework important for meeting patient needs were not covered by the functionality in any of the ICT tools.

**Conclusions:**

This study shows a lack of ICT tools developed and evaluated for supporting self-management of MACI patients. Our analytical framework was a valuable tool for providing an overview of how the functionality of these tools matched patient needs. There are a number of important areas for MACI patients that are not covered by the functionality of existing tools, such as support for interpersonal interactions and relationships. Further research on ICT tools to support self-management for patients with MACI is needed.

## Introduction

Information and communication technology (ICT) is a means to cope with the increasing number of patients with chronic diseases in our aging society [1]. For individuals with chronic illness affecting cognitive capacities either directly (eg, dementia) or indirectly (eg, diabetes), ICT has become a fundamental part in their daily lives by providing a wide range of useful services and tools to use at home, work, or anywhere else [2-5].

Intensive research is ongoing regarding ICT support for patients with moderate or severe cognitive impairments. One group that has not received much attention, however, is people with mild acquired cognitive impairments (MACI).

### Mild Acquired Cognitive Impairments

The new term *MACI* is used to differentiate patients with mild cognitive impairments (MCI) after acquired brain injury, such as traumatic brain injury (TBI), stroke, or other medical conditions or treatments, who are expected to reach a stable cognitive level over time from patients with a slowly deteriorating cognitive impairment, such as Alzheimer disease or schizophrenia [6].

The clinical definition of MACI is in line with the American Congress of Rehabilitation Medicine Special Interest Group on Mild TBI definition of mild TBI [7]: minor motor dysfunction/no motor dysfunction; appear to function well in social situations occasionally requiring support; may have a number of different cognitive disabilities, mostly within the area of attention, concentration, and memory; and may have a number of concomitant emotional problems. In order to be classified as having MACI a patient must meet the following 3 criteria: (1) the patient fits the general definition of having MCI, (2) the patient acquired this MCI as the result of a known medical condition, and (3) the patient’s cognitive state is expected to improve over time with treatment.

The largest etiological groups within MACI are patients with TBI, stroke, and brain injuries [8]. Each year, more than 1.5 million people in the United States suffer from TBI [9]. Mild TBI and concussion are the most frequent combat-related injuries. Brain injuries are also common at all levels of athletic competition and have been noted as a serious long-term health problem for retired professional American football players [10,11]. Although the majority of people with mild TBI resume normal functioning fairly quickly, approximately 5% to 15% report persistent cognitive and emotional symptoms [12,13].

Mild cognitive disability is a significant health problem and can result from a number of conditions. It may result in problems performing daily functions, such as reduced efficiency and reduced pace when performing activities. Problems may be persistent and decrease the overall effectiveness of the patient in the performance of routine activities of daily living, while also decreasing their capacity to adapt to novel or problematic situations [14,15].

The initial symptoms of mild TBI also apply to MACI patients and include dizziness, nausea, and impaired concentration that will typically decline during the first 3 months after the injury [16,17]. However, subgroups of patients develop persistent symptoms [18]. Patients can have multiple cognitive and/or behavioral and emotional disabilities, such as depression, low self-esteem, anxiety, lack of initiative, inability to maintain previous work pace, cognitive problems, and poor stress tolerance [19]. For this group, daily life becomes a challenge and the condition brings reduction in life satisfaction [20].

### Treatment and Self-management of Mild Acquired Cognitive Impairments

Patients with MACI are often of working age and can have quite complex and challenging problems; therefore, it is hoped that with the right tool, strategy, and treatment these patients may return to normal life and work. There are a number of challenges in treating these patients. Treatment strategies intended for moderate and severe acquired brain injuries are irrelevant for patients with MACI [13]. For example, the technologies developed to support MCI patients have focused primarily on Alzheimer disease and related problems, such as dementia. Treatment for MACI patients is quite different from those with moderate or severe injuries or Alzheimer disease for a variety of reasons. One issue is that patients with MACI need to be treated to handle a wider variety of situations than patients with moderate or severe injuries or Alzheimer disease. For example, they often need help to deal with interpersonal-emotional impairments, social situations, the work context, and with productivity-related skills [6,13]. Patients with MACI also do not have observable disabilities, such as motor and speech problems.

Treatment of MACI focuses on regaining lost skills and learning ways to compensate for lost abilities to allow patients to function well in all appropriate contexts and situations. For these reasons, the treatment options are also quite varied, and patients generally need individualized programs tailored to their capabilities, backgrounds, and interests. Treatment programs deliver assessment and reassurance by cognitive rehabilitation and stress management, and assist patients to return to work [21]. Studies have also shown that simple support in terms of education and group therapy appeared to provide extensive help for individuals with MACI with respect to their individual conditions and disabilities [21].

Because the goal of MACI treatment is often to help the patient become more independent and manage different life situations more effectively, an important aspect of the treatment is support for self-management. Self-management can be defined as “the individual ability to manage the symptoms, treatment, physical and psychosocial consequences, and lifestyle changes inherent in living with a chronic condition and disability” [22]. In this paper, our target is how to utilize ICT to enable the health care system so that it can support patients with MACI in self-management, for example, by recommending and/or providing the appropriate ICT tools to patients that can assist them. Self-management is a broad concept; therefore, the development of tools that support self-management must be conducted from a broad perspective [23]. Self-management programs have to emphasize the patients’ central roles in managing their illness and include both the medical and social aspects of living to manage a long-term chronic condition [24].

New possibilities are offered by ICT to enhance treatment, including support for group therapy and improved individual follow-up of rehabilitation support for optimal self-management, where individually adapted information and self-management tools can be combined with the integrated knowledge obtained within the framework of group treatments. Currently it is unclear what the best strategies are to support treatment of MACI with ICT. There are no concrete design guidelines that can aid designers in the development of new ICT tools to support patients with MACI in self-management and treatment.

The goal of this paper is to provide a contribution to the direction of future research on ICT tools that can be used by health care professionals who are seeking to assist MACI patients in self-management. The development of these tools will require multiple perspectives to be considered, including the perspective of the patient in managing and coping with their condition, and of the health care workers who treat the patient and will need to understand the potential of these tools and how to recommend them to specific patients.

We present an overview of studies reporting on the design and/or evaluation of ICT tools for assisting MACI patients in self-management. We also provide an analysis of the features of these tools using the International Classification of Functioning, Disability and Health (ICF) checklist [25] as a framework because it is frequently used by MACI rehabilitation professionals and medical experts for clinical assessment of MACI patients. Since the ICF checklist is comprehensive, we used a subset of the most common and important problems for MACI patients, determined by an MACI expert located in Sweden (AB) for our analysis.

Specifically, we will explore the following research questions: (1) What functionality has already been explored and/or evaluated regarding ICT tools that can be used to assist MACI patients in self-management? (2) What level of evidence exists that this functionality can provide benefits for MACI patients? (3) What gaps exist with respect to the functionality and the assessment framework used by rehabilitation professionals treating MACI patients?

We seek to aid health informatics by clarifying what functionality should or should not be recommended for assisting patients with MACI in self-management, what functionality appears promising but needs further evaluation before clear recommendations can be made, and what functionality may have been ignored in previous studies and should be targeted in design studies of future ICT tools to assist with MACI.

## Methods

### Review of the Literature

A review of the existing literature about available assistive ICT for people with MACI was conducted. This study was based on a review of the scientific literature published between 1995 and 2011 and retrieved between June and September 2011. The sources of the literature were the following electronic databases: MEDLINE (PubMed), Association for Computing Machinery (ACM) Digital Library, ScienceDirect, Ovid, Physiotherapy Evidence Database (PEDro), SpringerLink, ISI Web of Science (Science Citation Index Expanded), and the Institute of Electrical and Electronics Engineers (IEEE) Xplore Digital Library. Table 1 shows the inclusion and exclusion criteria.

**Table 1:** Inclusion and exclusion criteria for literature review of information and communication technologies (ICT) used for minor acquired cognitive impairments (MACI).

Criteria	Study characteristics	Study participants
Inclusion	Original articles	Mild/moderate cognitive impairments and dysfunctions
	English language	Mild/moderate acquired cognitive impairment and dysfunction
	Adult participants only	Severe injury but the mild/moderate outcome after certain period of time
	Where the technology was either created, evaluated or applicable for MACI patients	Non-progressive diseases
	Studies focused on technologies and tools that are developed to support patients’ self-management (involving patient’s responsibility for managing some aspects of their condition together with care professionals)	
Exclusion	Conceptual frameworks and literature reviews	Severe cognitive impairments and dysfunctions
	Studies focused on technologies that are developed for patients with a more severe cognitive decrease than for MACI patients	Aphasia
	Studies focused on technologies and tools that are developed for assessment and diagnostic purposes	Alzheimer disease
	Studies that included participants with mild cognitive impairments but with severe physical dysfunctions	Schizophrenia
		Psychotic disorder
		Developmental cognitive disabilities

Since MACI is a new term, it was not possible to rely on using it alone as a keyword. Thus, we expanded our search terms to include more broad cognitive and traumatic impairments to see if studies on these issues also included technologies relevant for MACI patients. The search terms *cognitive impairment*, *mild cognitive impairment*, *mild acquired cognitive impairment*, *traumatic brain injury*, *mild traumatic brain injury*, *mild head injury*, *mild acquired brain injury*, *memory disorder*, *concussion*, *post-concussive*, and *mild acquired cognitive dysfunction* combined with *assistive technology*, *informatics*, and *information technology* were used (55 search phrases in total). All citations were imported into reference management software (Endnote X4) to manage bibliographies and references and to remove duplications. The software also helped to identify and follow the authors who published relevant articles in the field.

Using the inclusion and exclusion criteria, the retrieved articles’ titles were read by the first author (AE) to eliminate the irrelevant articles. In the next phase, three authors (AE, JS, and SK) went through the abstracts and the full text if there was uncertainty about inclusion. The third author (AB) was also involved in the selection process in cases where there was ambiguity for the study from rehabilitation and medical point of view to make sure that the selection met the inclusion criteria. After final selection, the information was extracted from the full texts.

### Analytical Framework

One strategy for analyzing the features of ICT tools to determine how well they fit patient self-management needs is to conduct the analysis based on current evidence-based practice [26]. In the absence of well-established clinical guidelines for treatment of MACI patients, we used the ICF checklist to build an analytical framework to classify impairments that MACI patients may have [25]. The ICF offers an international and interprofessional scientific base for understanding and studying health and it has been used to understand the ability of ICT to assist at functional/cognitive, activity, and participation levels. The ICF checklist is comprehensive; therefore, we also conducted our analysis on a subset of the ICF checklist consisting of the most common and important problems for MACI patients as determined by an MACI expert located in Sweden (AB). The resulting framework was used to identify gaps between the features of existing technologies and the variety of impairments encountered by patients, from the perspective of health care professionals assisting with self-management of their condition.

The ICF also provides a checklist of major categories as a practical tool to elicit and record information on the functioning and disability of an individual [25]. These categories are related to the following components: body functions, body structure, activity and participation, environmental factors, and personal factors. This original checklist had to be filled by the extent (severity) of the impairments, difficulties, barriers, and facilitators. The extent of impairments for some MACI patients would be mild, for some moderate, and in rare cases, a severe impairment might be observed in certain aspects [25].

## Results

### Review of the Literature

**Figure 1:**
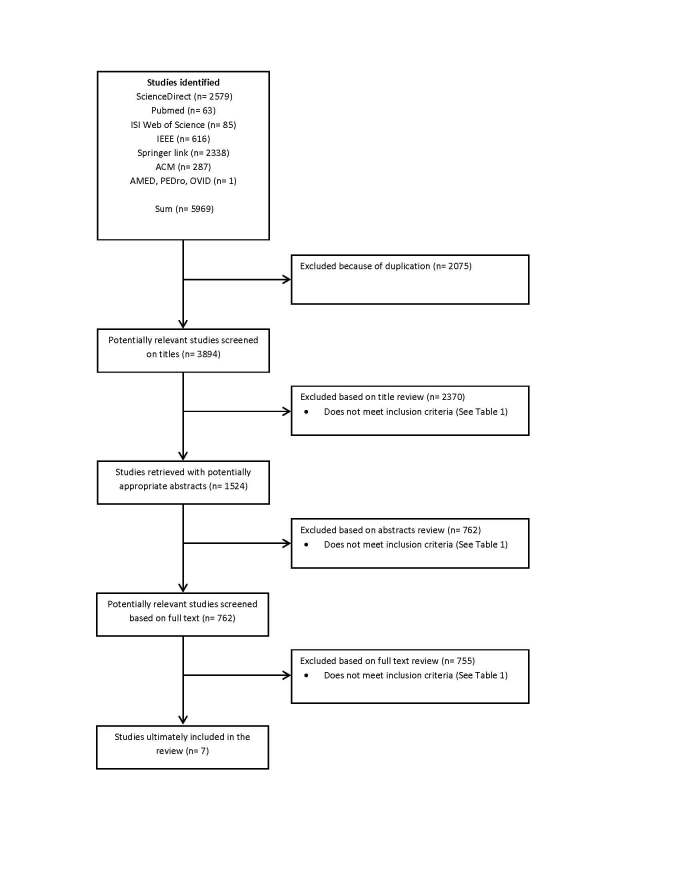
Flow diagram of the study selection process.

A total of 5969 publications were identified by initial keyword searches and 2075 were eliminated due to duplication. A further 2370 articles were excluded after reading the titles. The abstracts of 1524 articles were assessed. After exclusion of irrelevant articles, 762 articles were reviewed by reading the full text. In the end, 7 articles met the inclusion criteria. Because MACI is a new term, there is no standard way of bibliographic indexing for this filed yet, which required the authors to read the full text of a large number of articles. Figure 1 provides an overview of the journals and databases with numbers of selected and eliminated articles.

A total of 7 publications met the inclusion criteria. Table 2 displays the studies’ and systems’ names, countries of implementation or test, year of publication, type of publication, and references.

The analysis of identified articles showed that 2 of 7 articles (29%) were authored in the United States, 3 of 7 (43%) in Europe, and 2 of 7 (29%) in Asia.

**Table 2:** General details about the studies that met the inclusion criteria.

Title	Project	Year	Country	Type of articles	Journal/Conference
Designing a cognitive aid for the home: a case-study approach. [27]	Cognitive Aid for the Home	2003	United States	Conference proceedings	Association for Computing Machinery (ACM)’s Special Interest Group on Accessible Computing (SIGACCESS)
An interactive assistive system for prospective memory deficit compensation-architecture and functionality [28]	Mobile Extensible Memory Aid System	2003	Germany	Conference proceedings	SIGACCESS
A tele-cognitive rehabilitation platform for persons with brain injuries [29]	---	2006	Hong Kong	Conference proceedings	International technical conference of the Institute of Electrical and Electronics Engineers (IEEE) Region 10, the Asia Pacific Region
A cooking support system for people with higher brain dysfunction [30]	A cooking support system	2009	Japan	Conference proceedings	The ACM multimedia 2009 workshop on Multimedia for cooking and eating activities
Personal digital assistant (PDA) software aimed at improving workplace adaptation for people with cognitive disabilities [31]	Time and Task Manager (GTT)	2010	Spain	Conference proceedings	Computers Helping People with Special Needs
Computer based cognitive training for patients with mild cognitive impairment (MCI) [32]	Computer based cognitive training	2010	Greece	Conference proceedings	Pervasive Technologies Related to Assistive Environments
Electronic reminding technology following traumatic brain injury: effects on timely task completion [33]	Electronic reminding	2011	United States	Journal	The Journal of Head Trauma Rehabilitation

### Study Details

#### Study Type, Methodology, and Level of Evidence

As shown in Table 3, study types were distinguished as prototypes (early stage of system design that is built to test a process, concept, or human interaction to support user-focused research) or case reports (individual patient or group of patients have tested/evaluated the system or product). We found the level of evidence was very low. Of the 7 studies, 5 (71%) had poor methodologies and did not describe their design process and evaluations using a robust methodology. Table 3 illustrates that patients were involved in the design process in only 2 studies [27,31] and only 1 of them [27] used the actual user-centered design method [34,35]. Most of the studies were designed based on existing systems and guidelines and also considering the requests for such services.

**Table 3:** Study type and research method.

Reference	Study type	Research method	Design process
[27]	Prototype development	Case study (design and creation)	User-centered design
[28]	Case report	Exploratory research	Design process was not clear (based on existing electronic memory aid systems and requisites of a memory aid)
[29]	Case report	Exploratory research	Design process was not clear (cognitive rehabilitation strategies for problem solving training were implemented with flash communication software)
[30]	Case report	Case study (design and creation)	Design process was not clear
[31]	Case report (method is not clear)	Exploratory research	Document review, collection of information about tasks, problems and needs, prototyping, evaluation, redesign, implementation. (incremental development)
[32]	Case report	Exploratory research	Not described
[33]	Case report	Exploratory research	Not described

#### Participants (Patients)

Table 4 shows the demographic information of participants in the 7 studies. Most were patients with TBI and MCI, and the studies fit the criteria of patients with mild to moderate impairments. One of the identified studies had a more moderately injured patient as a user where the technology described had the potential to be used by MACI patients [29]. In another study, the patient group was somewhat unclear. The authors described their work as being relevant for dementia and problems affecting the elderly in the introduction section, but the methodology described participants in the study as 59 MCI patients [32].

**Table 4:** Demographic information about study participants.

Reference	Participants/system users	Number of participants	Severity of cognitive impairment
[27]	Mild traumatic brain injury	1	Mild/moderate
[28]	Persons with mild to moderate memory problems	9	Mild/moderate
[29]	People from Hong Kong, aged from 18 to 55, demonstrated basic attention and communication abilities, had gone through inpatient euro-rehabilitation, were medically stable	25	Mild/moderate
[30]	39-year-old female, aphasic with cognitive and memory disorders, often had difficulty with multistep tasks	1	Moderate
[31]	Workers with mild cognitive impairment	8	Mild cognitive impairment
[32]	Mild cognitive impairment patients	59	Mild cognitive impairment/dementia
[33]	Traumatic brain injury patients and self-determined complaints of memory impairment	36	Mild/moderate

### Study Features and Functionality

The development and implementation of assistive technologies in health care is usually intended to improve medical care and self-management [36]. The targeted studies were selected based on improving patients’ independence and supporting their self-management. After initially reviewing the studies, there appeared to be some trends in the functionality included in the tools. The authors divided this functionality into the following subcategories as reflected by these trends: improving independence, memory, problem solving, working, and task completion.

All *stationary devices* were personal computers and *mobile devices* were smartphones, personal digital assistants (PDAs) [37], and/or wearable devices. There was one special prototype with its own hardware design used as a digital frame. Considering the number of mobile systems, a large percentage of the systems’ input methods were through touch screens, but also buttons, PC input devices, and, in one case, a Nintendo Wii remote [30] (See Table 5).

**Table 5:** Study features and functionality.

Reference	Functionality	Improvement aim	Type	Setting	Technology	Interaction type	Component hardware type
[27]	Increase individuals’ functional independence in the home environment by providing time and location based prompts	Independence	Memory aid	Mobile	Mobile-based	Display (seeing)	A display that can be mobile or mounted on wall
[28]	To support patients with deficits in the prospective memory after a brain injury	Memory	Memory aid	Mobile	Mobile and Web-based	Touch	Mobile
[29]	Problem-solving skill straining	Problem-solving skills	Rehabilitation	Stationary devices	Web-based	PC input devices	PC
[30]	Cooking support	Activities of daily life, Undertaking multiple task	Education and training	Stationary devices	Computer based	Nintendo Wii remote	PC
[31]	Learning support, acquiring job skills, risk prevention	Support in areas such as learning tasks, acquiring job skills, risk prevention	Memory aid	Mobile	Mobile-based	Touch	PDA
[32]	Verbal-arithmetic-logic-spatial and memory exercises	To investigate the effectiveness of a computer based training on visual spatial abilities, visual attention, executive function and visual memory	Memory aid	Stationary devices	Computer based	PC input devices	PC
[33]	Producing higher rates of timely task completion	Timely task completion	Memory aid	Mobile	Mobile-based	Touch	PDA

#### Analysis of Functionality Based on Analytical Framework

**Figure 2:**
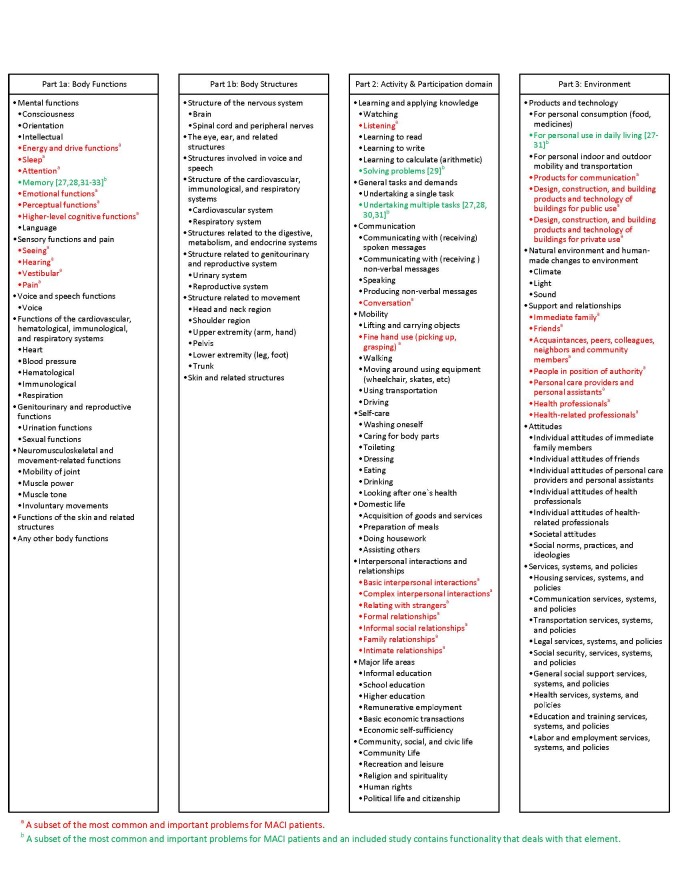
International Classification of Functioning, Disability and Health (ICF) checklist.

All identified studies were analyzed based on the analytical framework described previously in the methods section. The identified studies covered a few impairments from the ICF checklist for MACI patients, but most of the items in the ICT checklist were not addressed by the functionality of any of the systems that have been published. Figure 2 shows the ICF checklist and indicates which areas on the checklist correspond with the functionality of tools included in the study. The colored elements are the subset of the most common and important problems for MACI patients. The reference number next to some of the elements (green items) indicates which of the included studies contain functionality that deals with that element.

## Discussion

One of the most salient findings of the review portion of the study was that there is a general lack of published studies that report on the use of ICT to support self-management for MACI patients. The number of relevant articles found was very low (7 studies). The small number of studies that met the criteria for the review was not a result of a general lack of focus within the research community on studying ICT tools to aid patients with cognitive impairment. The problem was rather that a high number of studies reporting on ICT tools for self-management of patients with cognitive impairments were developed for patients with severe impairments, Alzheimer disease, and/or age-related deficiencies. Patients with MACI have different needs than these patients and have received far less attention from the research community.

### Functionality Included in the Tools

With respect to the functionality of the tools that did meet the review criteria, we found the ICF checklist and a subset of the most common and important problems faced by MACI patients, identified and used by experienced rehabilitation professionals and MACI medical experts in Sweden, to be valuable as an analytical framework for investigating how their functionality meets patient needs and treatment options from the perspective of health care professionals that would assist them in self-management. All of the tools that met the inclusion criteria were focused on helping to support patients in managing daily activities, and all of them met the common and important problems criteria. This suggests that the line of research of tools to support MACI has been focused on highly relevant problems. More specifically, all of the studies helped aid patients with at least one of 3 things: (1) memory deficits [27,28,31-33], (2) undertaking multiple tasks at the same time [30], and (3) problem solving [29].

Although our analysis included health care professionals’ perspectives on patients’ needs by including MACI experts’ experience and knowledge to identify relevant patient problems on the ICF checklist, in the future it would be interesting to improve the analytical framework by collecting data directly from patients to see whether this information differs from that provided by MACI experts. This could be useful for further analyzing ICT self-management and treatment tools and for other aspects of MACI treatment because this is a new area that needs further development. It could also be used as a basis for considering how to design tools that could be used for self-management of patients outside of the context of collaboration with health care professionals that we have targeted in this paper.

### Study Methodology and Evidence of Benefit

Although the studies focused on issues that seemed important for MACI patients, a general limitation with the studies is that they did not report evaluations using a robust methodology that could provide a high degree of evidence on the usefulness of the tools investigated. We had hoped to be able to provide a discussion about whether or not the tools that were developed thus far were beneficial for patients. However, the quality of the studies was so low that it was not possible to do that at this time.

For example, only 1 study reported an evaluation of a system that included a control group and the findings were statistically significant in favor of the group using the tool versus the group that did not use the tool [32]. The rest of the articles either reported a case study or were exploratory in nature, and thus had a focus on identifying design issues rather than on providing clear evidence of benefits to patients for the tool being studied. Although the preliminary results do seem positive for the tools included in these studies, additional studies are needed to determine benefits for patients.

Another methodological limitation of the reported studies is that they often did not describe their own design process very clearly. In most cases, we could not comprehend the entire design process utilized. However, one of the studies did report the utilization of user-centered design methods by developing a prototype based on patients’ preferences as identified during participatory design, and the system’s capabilities [27]. This is consistent with suggestions that design processes for ICT services that will be utilized by patients should require users to be involved in the design process. Representative users should actively participate, early and continuously throughout the entire development process and throughout the system lifecycle [38,39]. User-centered design will address the challenges about design approaches in health informatics, usability problems, visions for further development, and the necessary improvements in practical user-centered guidelines for designing ICT tools [40]. In the future, studies should clearly report on the design methodology and involve the users in the design process.

### Gaps in the Functionality of the Tools

One of the goals of this paper was to identify unexplored functionality that could be useful for supporting MACI patients with self-management by finding issues on the subset of common and important problems taken from the ICF checklist that did not appear in any of the studies. A total of 34 items appeared on the subset, but only 4 of these items (13%) were covered in the functionality of the tools identified during the study. Therefore, the study indicates that there is much progress still to be made in the area and that new tools are worth exploring in order to expand the number of different ways that MACI patients can be supported with ICT.

The development and evaluation of additional tools provides the possibility to fill in the gaps for impairments noted in the ICF checklist that are not covered by the existing tools. The items on the list are also quite general and the MACI patients’ impairments are very individual. This means that not all patients with an impairment that qualifies under a specific ICF subcategory will be able to obtain benefits from all ICT tools targeting that category.

Areas on the subset of common and important problems that are not covered by the tools in the study can be viewed as interesting areas for future investigations. One issue that seems highly relevant, for example, is that none of the tools supported interpersonal interactions and relationships although family support, social interaction, and relationships with friends, which are important issues for patients with MACI [13]. It may be interesting to explore the usage of the Internet and social media for these purposes [41]. Also, the long-term information exchange between patient, families, and caregivers, and the long-term effect of using such technologies and follow-ups are unexplored areas. None of the studies investigated utilized Medicine 2.0 [42] (ie, the use of specific Web tools for supporting and personalizing the health care collaboration and education) and these are interesting new areas that are being utilized to support social needs of patients.

### Additional Issues

In addition to looking at how the individualized needs of MACI patients not addressed in the reported studies can be supported in the future, there is also the opportunity to benefit from investigating how to combine and/or configure different tools to meet the individualized needs of patients. Although it may be possible to address a variety of impairments associated with many different elements on the ICF checklist by one multifunctional tool, it is likely that multiple useful tools will be developed and evaluated independently, and that guidelines for how best to combine different tools to meet different patient needs will need to be developed.

It is possible that there will be a large role to play in this process through the use of open market tools rather than new tools specifically developed for MACI patients. The impairment of MACI patients is usually limited and they are capable of using computers, smartphones, and the Internet on their own. There also is a wide range of different applications available on open market platforms, such as smartphones and tablets, that might be able to address many of the challenges faced by MACI patients. Thus, it is interesting to see if providing patients with combinations of open market tools strategically selected to serve their individualized needs will provide benefits.

In addition to thinking about how the functionality of tools matches with the ICF checklist and the subset of common and important problems, there are some other notable issues that can be taken from the review that can help to guide future research. One issue, for example, is that the advancement of development tools and platforms now makes it possible for developers and software/hardware designers to computerize existing rehabilitation approaches. However, recent articles have not exploited this opportunity. It would be interesting to conduct studies that focus on how existing rehabilitation frameworks can be adapted to ICT tools to provide patients with improved self-management possibilities.

### Limitations

This research provided an overview of peer-reviewed literature on this topic and the required design and direction for future research. However, this research was limited to all published articles before September 2011 because the reading of a large amount of full text was needed due to inconsistent bibliographic indexing of this fairly new field of research. We also might have missed assistive technologies, system developments, and implementations that were not published in the scientific journals, as well as the most recent developments.

### Conclusion

In our review of ICT tools that can be used for health care professionals to support self-management of MACI patients, only 7 relevant studies were found. The existing studies provide an overview of some ways in which patients can be aided with memory problems, problem solving abilities, and handling multiple tasks. However, further studies are needed on how to support patients with these problems because the methodologies used for evaluating these tools were insufficient to provide clear clinical recommendations. The existing studies also did not describe their design methodology in detail; future studies could provide additional value by reporting such details.

The functionality of all of the tools that met the inclusion criteria for the review fell within the subset of the ICF checklist consisting of the most common and important problems for MACI patients. This provides some validation for using these criteria as an analytical framework because all the other groups independently ended up focusing on the same problems that have been identified by the medical experts. Further efforts to refine and standardize these criteria are needed because MACI is a new term and the criteria were developed from only a few experts in one country.

In addition to further exploring tools with functionality like those in the studies identified in the review, the list of common and important problems is useful for highlighting gaps where new tools can be developed to potentially aid patients in ways not supported by existing tools. There are a large number of items on the list that are not covered by existing tools. Therefore, there is a large opportunity for the research community to investigate improved support for MACI with ICT. In addition to the development of custom tools, evaluation of open market tools would be valuable, as well as investigating how to combine multiple tools to provide individualized support for patients.

## Abbreviations

ICF: International Classification of Functioning, Disability and Health
ICT: information and communication technology
MACI: mild acquired cognitive impairment
MCI: mild cognitive impairment
TBI: traumatic brain injury
